# Protective Effect of Curcumin on Acute Airway Inflammation of Allergic Asthma in Mice Through Notch1–GATA3 Signaling Pathway

**DOI:** 10.1007/s10753-014-9873-6

**Published:** 2014-04-06

**Authors:** Lei Chong, Weixi Zhang, Ying Nie, Gang Yu, Liu Liu, Li Lin, Shunhang Wen, Lili Zhu, Changchong Li

**Affiliations:** 1 Department of Pediatric Pulmonology, the Second Affiliated Hospital and Yuying Children’s Hospital of Wenzhou Medical University, Xue-yuan West Road 109, Wenzhou, 325000 Zhejiang China; 2 Department of Pediatric 3 Ward, Affiliated Taihe Hospital of Hubei college of Medicine, South People Road 32, Shiyan, 442000 Hubei China

**Keywords:** curcumin, asthma, airway inflammation, Notch, GATA3

## Abstract

Curcumin, a natural product derived from the plant *Curcuma longa*, has been found to have anti-inflammatory, antineoplastic and antifibrosis effects. It has been reported that curcumin attenuates allergic airway inflammation in mice through inhibiting NF-κB and its downstream transcription factor GATA3. It also has been proved the antineoplastic effect of curcumin through down-regulating Notch1 receptor and its downstream nuclear transcription factor NF-κB levels. In this study, we aimed to investigate the anti-inflammatory effect of curcumin on acute allergic asthma and its underlying mechanisms. 36 male BALB/c mice were randomly divided into four groups (normal, asthma, asthma+budesonide and asthma+curcumin groups). BALF (bronchoalveolar lavage fluid) and lung tissues were analyzed for airway inflammation and the expression of Notch1, Notch2, Notch3, Notch4 and the downstream transcription factor GATA3. Our findings showed that the levels of Notch1 and Notch2 receptors were up-regulated in asthma group, accompanied by the increased expression of GATA3. But the expression of Notch2 receptor was lower than Notch1 receptor. Curcumin pretreatment improved the airway inflammatory cells infiltration and reversed the increasing levels of Notch1/2 receptors and GATA3. Notch3 receptor was not expressed in all of the four groups. Notch4 receptor protein and mRNA expression level in the four groups had no significant differences. The results of the present study suggested that Notch1 and Notch2 receptor, major Notch1 receptor, played an important role in the development of allergic airway inflammation and the inhibition of Notch1–GATA3 signaling pathway by curcumin can prevent the development and deterioration of the allergic airway inflammation. This may be a possible therapeutic option of allergic asthma.

## INTRODUCTION

Turmeric is a perennial plant cultivated throughout the tropics, especially in India, China, and Indonesia. It is used in a number of culinary preparations and curcumin gives the curry its unique flavor and color. Curcumin, isolated from the rhizomes of turmeric, is the main active component and has been found to have many biological effects such as anti-inflammation, anti-oxidization, free radical removal, and anti-cancer [1–3].

Asthma is an airway inflammatory disease, characterized by lung eosinophilia, lymphocytes and neutrophils infiltration, mucus hypersecretion and airway hyper-responsiveness (AHR) to inhaled allergens [4]. Many inflammatory cells such as T lymphocytes, eosinophils and mast cells play a defensive role in the pathogenesis of asthma, in which the most important element is the activation of CD4^+^T cells. CD4^+^T cells can be divided into Th1 and Th2 types. Th2 reactions result in allergic responses, such as asthma [5].

Notch signaling pathway is an evolutionarily conserved cell-to-cell communication cascade that was originally identified as a pleiotropic mediator of cell fate in flies. It plays a critical role in maintaining the balance between cell proliferation, differentiation, and apoptosis [6]. Guo and colleges [7] have found that the level of Notch1 was significantly higher in asthma mice and Notch1 signal may play an important role in the pathogenesis of asthma by its involvement in Th1/Th2 differentiation.

A previous study demonstrated that curcumin might attenuate allergic airway inflammation and hyperresponsiveness in mice through inhibiting NF-κB [8] and its downstream transcription factor GATA3 [9]. Another research found that the down-regulation of Notch signaling and its downstream NF-κB by curcumin may be a novel strategy for the treatment of patients with pancreatic cancer [10]. These results indicated that curcumin might have a beneficial effect on asthma through inhibition of the Notch–GATA3 signaling pathway. Our aim in the present study was to examine the effect of curcumin in ovalbumin (OVA)-induced airway inflammation in a mouse model of asthma.

## MATERIALS AND METHODS

### Acute Asthma Model Set-up

A total of 36 specific pathogen-free male BALB/c mice, 4 to 6 weeks old and weighing 18–22 g and obtained from Shanghai Slac Laboratory Animal Center (Shanghai, China) were used in this study. All animals were kept in the animal center of Wenzhou Medical University and acclimated for at least a week before the experiments. All care and treatments of the experimental animals were in strict accordance with the recommendations in the Guide for the Care and Use of Laboratory Animals of the National Institutes of Health. The Committee on the Ethics of Animal Experiments of the Wenzhou Medical University approved the protocol (Permit Number: wydw 2009-0002).

The mice were randomly divided into four groups (normal, asthma, asthma+budesonide and asthma+curcumin groups). On days 1 and 15, experimental groups of mice were sensitized by i.p. injection of suspension containing 10 μg OVA (Sigma, USA) and 20 mg Al (OH)_3_ gel. From days 25 to 32, they were challenged in an inclosed environment filled with OVA aerosol (1 mg/ml) for 30 min every day. The curcumin treatment group received curcumin (Sigma, USA) fluid (200 mg/kg) from mouth before OVA challenge until the last challenge and the budesonide group used budesonide aerosol (0.5 mg/ml). The control group received normal saline on the same schedule.

### Bronchoalveolar Lavage and Lung Tissues Handling

All mice were narcotized by chloral hydrate (400 mg/kg) and sacrificed within 24 h after the last challenge. After sacrifice, mice were ligated one side of the bronchus and bronchoalveolar lavage fluid (BALF) was collected by flushing one side of the lungs with three separate normal saline through the trachea. The rate of recovery was more than 80 %. Different cell counts were determined for each BALF sample using a cytometer to be 1 × 10^4^ cells/ml. The nonlavaged side of lungs were stored in the Ultra-low temperature freezer or fixed in 10 % formalin and then made into 5-μm-thick paraffin-embedded tissue sections.

### Histology

Lung paraffin sections were stained with hematoxylin and eosin (HE). The degrees of allergic airway inflammation were scored by another person who was blinded for treatment of mice and were assessed according to the following histologic grading system (scored 0–4): absence of peribronchial inflammatory cells; a few scattered peribronchial inflammatory cells involving less than 25 % of the circumference of the bronchus; focal peribronchial inflammatory cell infiltrate not completely surrounding a bronchus (i.e., involving approximately 25–75 % of the circumference of the bronchus); one definite layer of peribronchial inflammatory cells completely surrounding a bronchus; two or more layers of peribronchial inflammatory cells completely surrounding a bronchus. In each lung section, the mean peribronchial inflammatory score was calculated by adding the scores of all the individual bronchioles in the lung section and dividing this score by the number of bronchioles present in the lung section [11].

### Immunohistochemistry

Immunohistochemistry was carried out using SP two-step IHC detection reagent (ZSGB-Bio, China). The primary antibodies were goat polyclonal anti-Notch1 (1:150; Santa Cruz Biotechnology, USA), rabbit polyclonal anti-Notch2 (1:400; Millipore Corporation, USA), goat polyclonal anti-Notch3 (1:50; Santa Cruz Biotechnology, USA) and rabbit polyclonal anti-Notch4 (1:100; Santa Cruz Biotechnology, USA). All sections were stained with DAB for a short time and counterstained with hematoxylin. Slices were examined and imaged with Olympus microscope (Olympus CX21FS1, Japan) with an attached digital camera using the same system settings for all samples. The brown colors in the slices were positive areas. The mean optical density (MOD) was then detected by Image-Pro Plus in all slices.

### RT-PCR

Total RNA was extracted using Trizol Reagent (Invitrogen Corporation, USA) according to the manufacturer's instruction. Two micrograms of RNA was reverse-transcribed to cDNA by incubating with reverse transcription reagents (Fermentas Corporation, USA) at 37 °C for 1 h. Primers specific for mouse Notch1, Notch2, Notch3, Notch4 and β-actin were as follows: for Notch1 the forward primer was TGGAGGTAGGTGCGAAGTG and the reverse primer was GGCAGCGACAGATGTATGAAG, for Notch2 the forward primer was CAACTGTGAGGTGGACAAAAATG and the reverse primer was TGTTCATACACGGCTTGGAGATA, for Notch3 the forward primer was TTCTCCTGTCGTTGTCTCCG and the reverse primer was GGCACTCATCTATGTCACTTTGG, for Notch4 the forward primer was CACCAGGCTTGGAAGGGAG and the reverse primer was GAAACCAGGACGGCAGAGG, for β-actin the forward primer was CGGGACCTGACAGACTACCTCAT and the reverse primer was CCACAGGATTCCATACCCAAGA.

The amplification protocol consisted of preheating at 94 °C × 5 min, followed by 32 cycles of denaturation at 94 °C × 30 s, annealing × 30 s and extension at 72 °C × 30 s. The PCR products were determined by agarose gel electrophoresis and analyzed by SMART Gel image analysis system.

### Western Blotting

The lung tissues were homogenized, incubated in lysis buffer, added with a protease inhibitor cocktail and then ultrasonic broken nucleus to obtain extracts of lung proteins. The samples were loaded to 10 % SDS-PAGE gels and transferred onto a PVDF membrane (Pierce Company, USA), then blocked with 5 % skim milk for 2 h. The blots were probed with the appropriate antibodies to assess the protein level of NICD1 (Epitomics, USA) and GATA3 (Santa Cruz Biotechnology, USA). The appropriate secondary antibody, conjugated to peroxidase and the enhanced chemiluminescence blotting system, was used for detection. The loading control was beta-actin.

### Statistical Analyses

All results were expressed as means ± SD and analyzed by one-way ANOVA, with SPSS 17.0. A *P* value <0.05 was considered statistically significant.

## RESULTS

### Effect of Curcumin on Acute Airway Inflammation in BALF

**Fig. 1:**
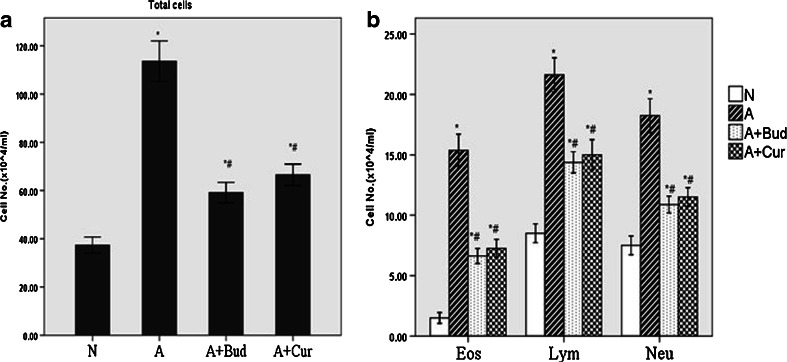
Curcumin inhibited inflammatory cells infiltration in BALF (*N* normal group, *A* asthma group, *A+Bud* budesonide treatment group, *A+Cur* curcumin treatment group). **a** Total cell counts in BALF. **b** Different cell counts (eosinophils, lymphocytes and neutrophils) in BALF. Data are means ± SD of eight mice per group. *P < 0.01 compared with the normal group; ^#^ *P* < 0.01 compared with the asthma group.

To evaluate the acute airway inflammation of OVA induced allergic asthma model, the total cell and different kinds of cell numbers in BALF were determined. Upon OVA sensitization and challenge, asthma group (A) mice show increased total cell influx in BALF compared to normal group (N) (*P* < 0.01). While in curcumin treatment group (A+Cur) and budesonide group (A+Bud) total cell influx were significantly decreased compared to asthma group (*P* < 0.01) but still higher than normal group (*P* < 0.01) (Fig. 1a). The trend of different kinds of cell numbers like lymphocyte (Lym), eosinophil (Eos) and neutrophil (Neu) were similar with the total cells (Fig. 1b).

### Effect of Curcumin on Airway Inflammatory Cells Infiltration in Lung Tissue

**Fig. 2:**
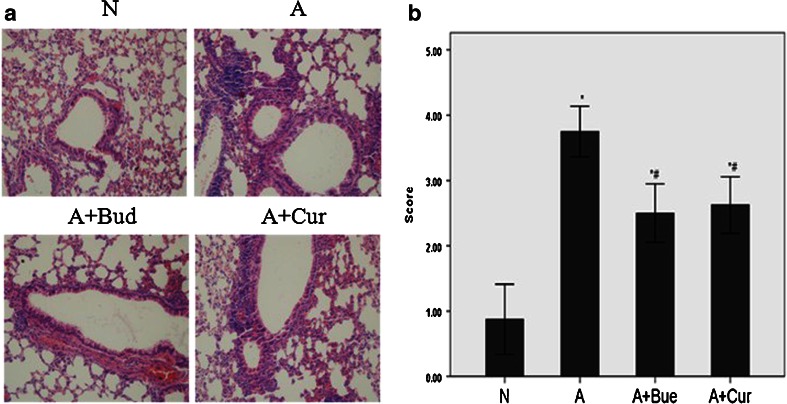
Curcumin attenuated acute airway inflammation in asthmatic mice. **a** Representative HE-stained (light microscopy, ×400) lung tissue slides of normal group, asthma group, budesonide treatment group and curcumin treatment group. **b** Semiquantitative pathology scores (described in Materials and Methods). Data are means ± SD of eight mice per group. *P < 0.01 compared with the normal group; ^#^ *P* < 0.01 compared with the asthma group.

HE-stained slides of lung tissue were scored for degrees of allergic airway inflammation in a semiquantitative fashion as described in “Materials and Methods” (Fig. 2a). OVA challenge resulted in airway inflammation in asthma group. After curcumin or budesonide treatment, the scores of airway inflammation were declined compared to asthma group (*P* < 0.01), corroborating the findings in BALF shown in Fig. 1 (Fig. 2b).

### Effect of Curcumin on Four Notch Receptors

To determine the role of curcumin in Notch receptors, all of the four Notch receptors protein and mRNA were detected.

#### Notch1 and Notch2 receptors

**Fig. 3:**
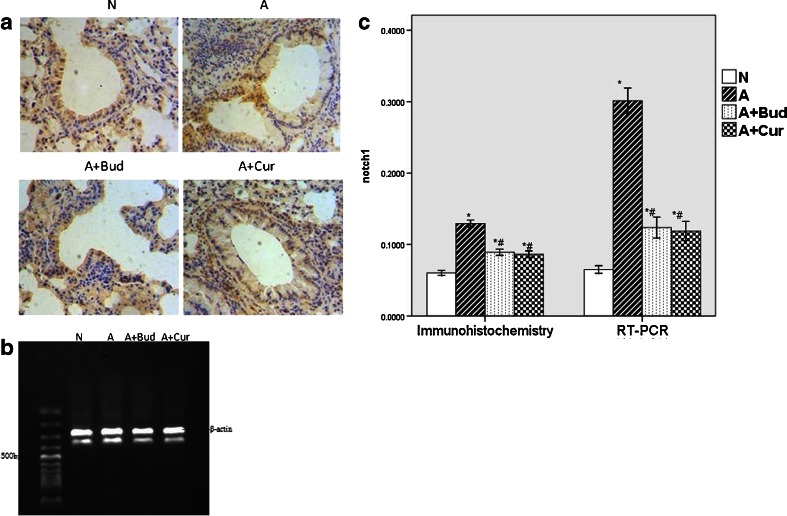
Curcumin decreased the expression of Notch1 receptor protein and mRNA. **a** Notch1 protein expression using immunohistochemistry staining (light microscopy, ×400). **b** Notch1 mRNA expression using RT-PCR. **c** The quantitative data of the protein and mRNA expression of Notch1 receptor. *P < 0.01 compared with the normal group; ^#^ *P* < 0.01 compared with the asthma group.

**Fig. 4:**
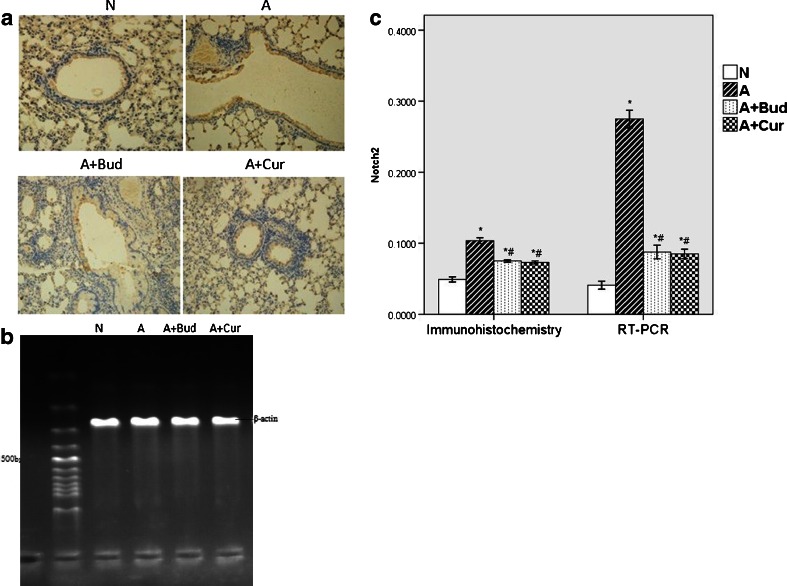
Curcumin decreased the expression of Notch2 receptor protein and mRNA. **a** Notch2 protein expression using immunohistochemistry staining (light microscopy, ×400). **b** Notch2 mRNA expression using RT-PCR. **c** The quantitative data of the protein and mRNA expression of Notch2 receptor. *P < 0.01 compared with the normal group; ^#^ *P* < 0.01 compared with the asthma group.

**Fig. 5:**
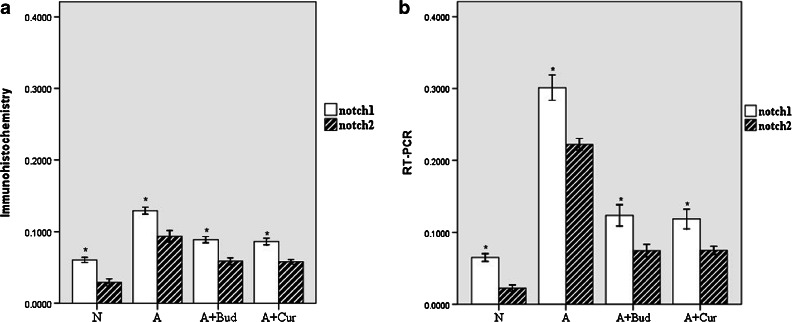
The expression of Notch2 receptor was lower than Notch1 receptor. **a** The quantitative data of the protein expression of Notch1 and Notch2 receptor. **b** The quantitative data of the mRNA expression of Notch1 and Notch2 receptor. *P < 0.01 compared with Notch2 receptor.

As shown in Fig. 3, the expressions of Notch1 receptor protein and mRNA were both significantly increased (*P* < 0.01) in asthma group compared with normal group. After treating with curcumin and budesonide, the levels of Notch1 receptor and Notch2 receptor proteins and mRNA were significantly decreased (*P* < 0.01) (Fig. 4). But the expression of Notch2 receptor was lower than Notch1 receptor (*P* < 0.01) (Fig. 5).

#### Notch3 Receptor

**Fig. 6:**
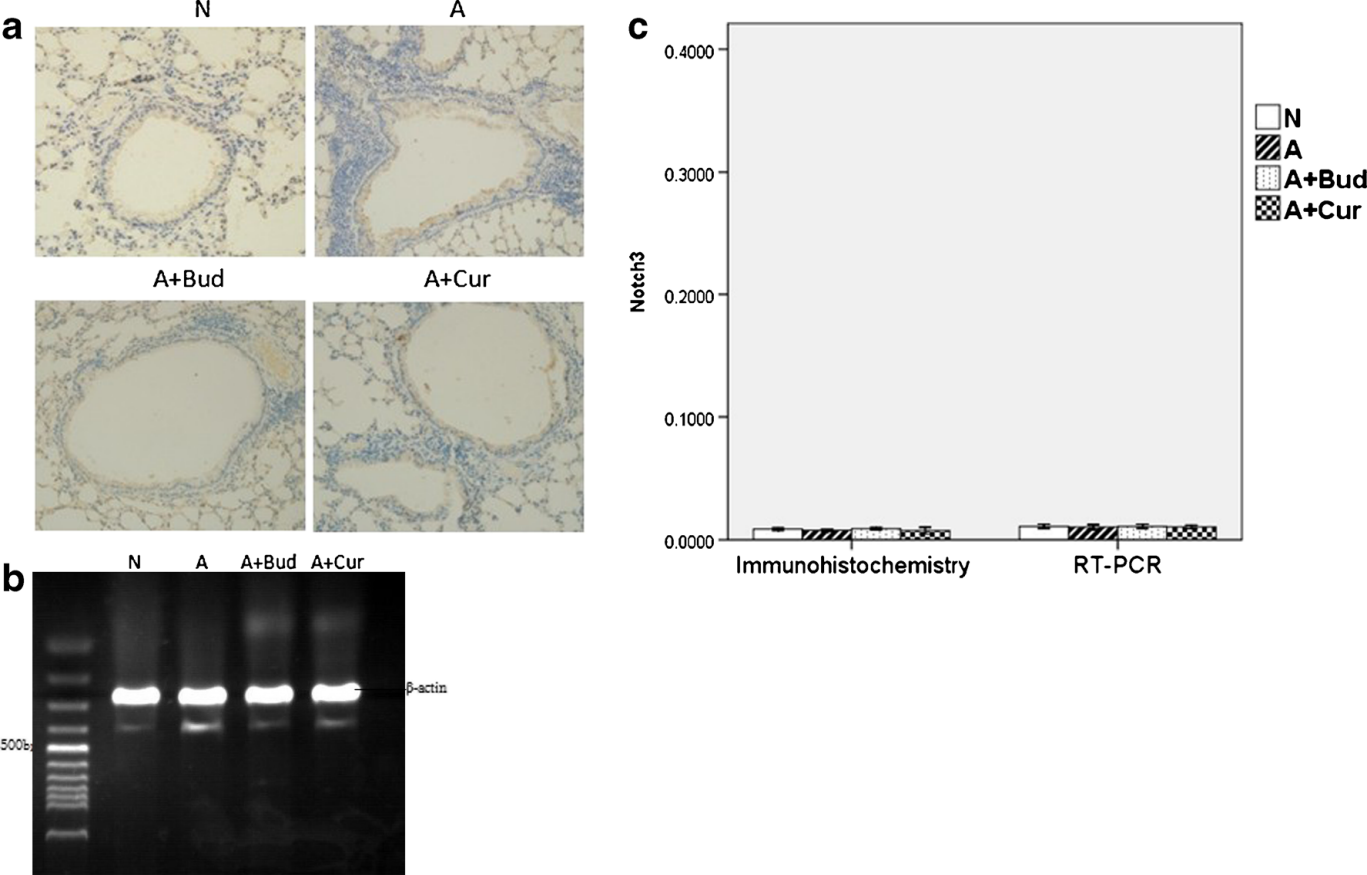
Notch3 receptor was not expressed in the four groups. **a** Notch3 protein expression using immunohistochemistry staining (light microscopy, ×400). **b** Notch3 mRNA expression using RT-PCR. **c** The quantitative data of the protein and mRNA expression of Notch3 receptor.

Mice in all four groups did not express Notch3 receptor, protein, or mRNA (Fig. 6).

#### Notch4 Receptor

**Fig. 7:**
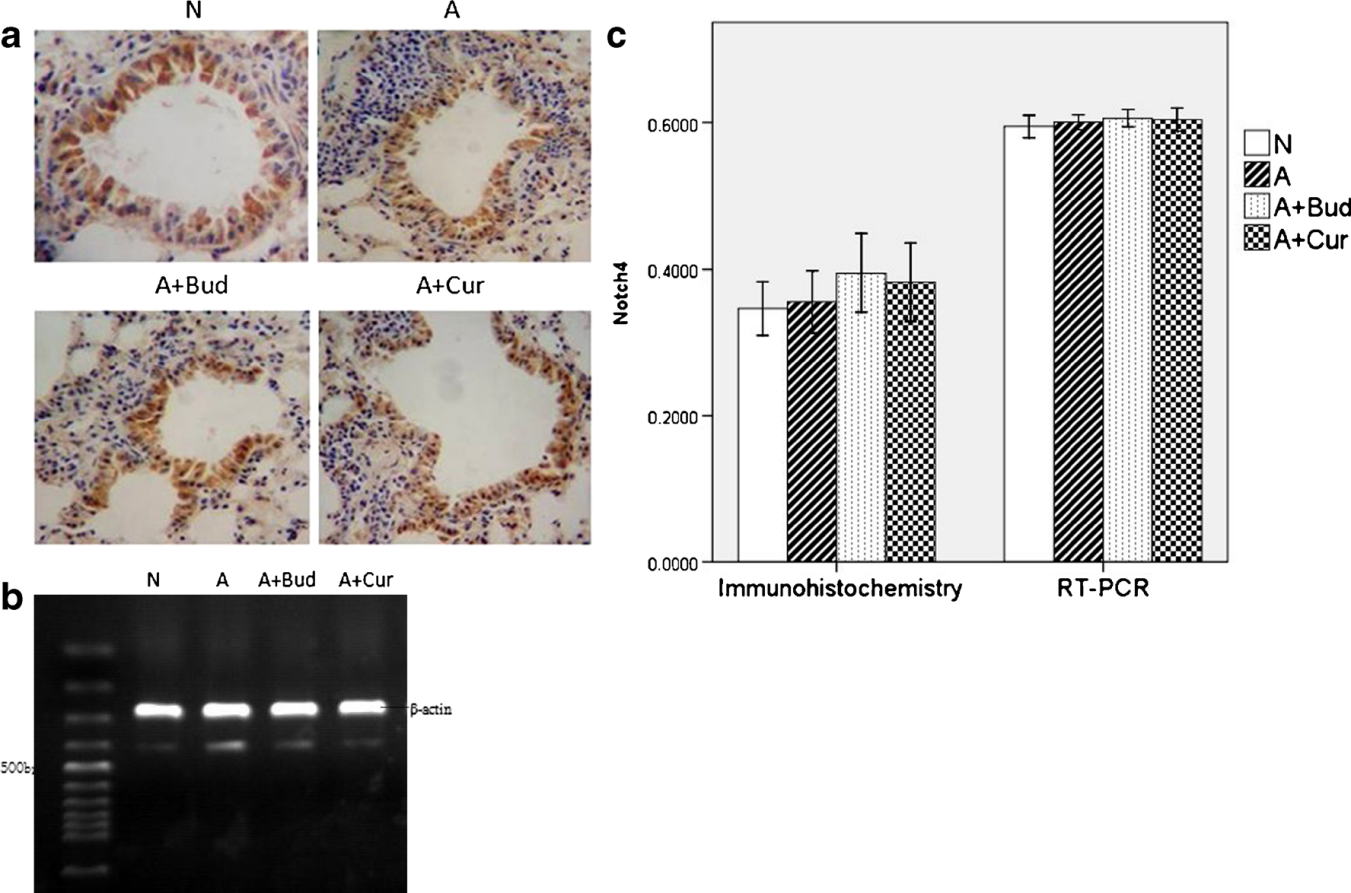
Curcumin did not affect the expression of Notch4 receptor. **a** Notch4 protein expression using immunohistochemistry staining (light microscopy, ×400). **b** Notch4 mRNA expression using RT-PCR. **c** The quantitative data of the protein and mRNA expression of Notch4 receptor.

The levels of Notch4 receptor protein and mRNA were very high. However, as shown in Fig. 7, there was no significant difference between each other among the four groups.

### Effect of Curcumin on Notch1 Signaling Pathway

**Fig. 8:**
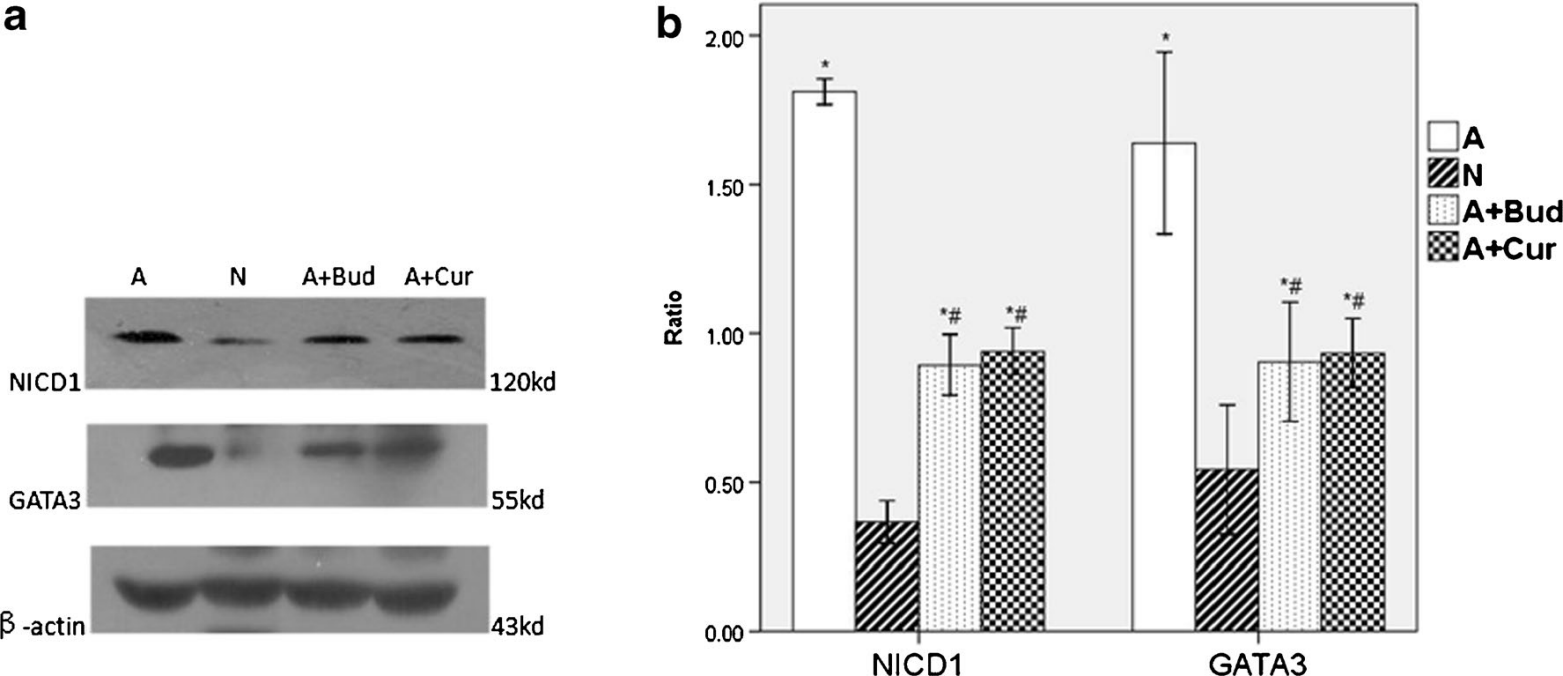
Curcumin inhibited the expression of NICD1 and its downstream transcription factor GATA3 protein. **a** The immunoblot analysis of NICD, GATA-3 andβ-actin. **b** The quantitative data of the protein expression of NICD1 and GATA3. *P < 0.01 compared with the normal group; ^#^ *P* < 0.01 compared with the asthma group.

Since curcumin showed inhibited effect on Notch1 receptor, we than detected the expression of NICD1, the active fragment of Notch1 receptor, and its downstream transcription factor GATA3 to determine whether Notch1 signaling pathway was associated with the inhibited effect of curcumin. From Fig. 8, we could find that the expressions of NICD1 and GATA3 protein were significantly increased in asthma group (*P* < 0.01) compared with normal group, but significantly decreased when treated with curcumin and budesonide (*P* < 0.01).

## DISCUSSION

Curcumin, a hydrophobic polyphenol derived from the rhizomes of turmeric, has been confirmed to have numerous therapeutic effects by modern scientific research [3]. Anti-inflammatory effect is one of the most important biological effects of curcumin [3, 12]. Some studies demonstrated that curcumin might attenuate allergic airway inflammation through NF-κB inhibition [8] and might be a novel therapy for pancreatic cancer through down-regulating the expression of Notch1 and its downstream transcription factor NF-κB [10]. So, could allergic airway inflammation of asthma be attenuated by curcumin through the inhibition of Notch signaling pathway?

Asthma is a disease of the airways characterized by chronic inflammation associated with AHR and airway wall remodeling. Airway inflammation is central to disease pathophysiology. The airway dysfunction may partly due to the release of potent inflammatory mediators and partly due to the remodeling of the airway wall [13, 14]. A large number of inflammatory cells such as T lymphocytes, eosinophils and mast cells and inflammatory mediators such as IL-4, IL-5, IL-9, IL-13, TSLP, CXCL10, CCL11 and RANTES infiltrated into lung tissues, gathered around the tracheal bronchus, and fluxed into the BALF [5, 15]. Our study found that in OVA-induced allergic asthma mice model, the numbers of total cells influx in BALF and the scores of airway inflammation were increased significantly compared with normal groups. The increasing of total cells was caused by the increasing of Eos, Lym and Neu, which was in accordance with the previous studies [16, 17]. All these results suggested that our asthma model was established successfully. And as expected, we also found that curcumin pretreatment significantly decreased the inflammatory cells infiltration in the lung tissues and BALF, which confirmed the anti-inflammatory effect of curcumin in allergic asthma.

Notch, a kind of transmembrane protein which was first found in flies, was ideally suited to precisely regulate cell–cell communication during development of complex tissues like the lung [18–20]. Recently, it has become increasingly clear that the Notch signaling plays important roles in the immune system such as asthma [6, 20, 21]. One potential role for Notch signaling in the immune system is promoting peripheral T cells activation, proliferation and cytokine production. There are four Notch receptors in mammals (Notch1–4), which are bound by five ligands (Delta-like1, 3 and 4 and Jagged 1, 2) [22]. Different Notch signaling may direct different Th cells proliferation. Interactions between DLL1/4 and Notch3 were described to promote a Th1-cell phenotype [23–25]. After exposure to Th2 cell stimuli, Notch1 and Notch2 would combine to ligand Jagged 1/2 to induce Th2 cell differentiation [26–28]. Since Th2 cells are the most important inflammatory mediators of allergic asthma [29], we speculate that Notch1 and Notch2 receptor will be increased in asthma group. And in the previous study, by inhibiting Notch signal in spleen CD4^+^T cells of allergic asthma mice, we have demonstrated *in vitro* that Notch signal plays an important role in asthma [30]. In this study, we demonstrated *in vivo* that Notch1 and Notch2 were indeed playing a positive role in asthma, but the effect of Notch2 was much lower than Notch1. Notch3 and Notch4 had no effect on asthma. We also found that in curcumin pretreatment groups, the levels of Notch1 and Notch2 receptors were significantly decreased compared to asthma groups though still higher than normal mice, which suggested that curcumin might attenuate allergic airway inflammation through inhibiting Notch signaling.

As described above, when exposure to a Th2 cell stimuli, Notch1 and Notch2 would be active and release intracellular domain NICD1 and NICD2 which would translocate to the nucleus and bind to transcription factor CSL on the promoter of Gata3 that ultimately leading to the production of Th2 cell cytokines IL4, IL5 and IL13 [6]. Other research studies also confirmed that GATA3 was necessary for Notch induced Th2 differentiation and identified an upstream Gata3 promoter CSL as a direct target for Notch signaling [31–33]. Besides, the results that NF-κB played a critical role in Gata3 expression and Th2 differentiation in allergic airway inflammation [9] suggested that NF-κB was one of the upstream factors of GATA3 which could regulate Th2 differentiation. Then we considered whether Notch–GATA3 signaling pathway was an important pathway in allergic asthma and whether it could be inhibited by curcumin. Since the expression of Notch2 receptor was much lower than Notch1 receptor, so we detected Notch1 intracellular domain NICD1 protein and its downstream transcription factor GATA3 protein expression in the four groups to explore whether curcumin could impair the airway inflammation by inhibiting Notch1–GATA3 signaling pathway. And we found that in asthma groups, the expressions of NICD1 and GATA3 proteins were significantly increased than normal groups. In contrast, pretreating with curcumin reversed the increasing levels of NICD1 and GATA3.

On the whole, our study proved the anti-inflammatory effect of curcumin on OVA-induced allergic asthma model by inhibiting Notch1–GATA3 signaling pathway. Therefore, curcumin may be considered as a potential agent for preventing asthma in the future. But before clinical application, there is still a long way to run and further and comprehensive studies are needed.
